# St. George's Hospital

**Published:** 1916-04-29

**Authors:** F. J. Frankau

**Affiliations:** Deputy Treasurer. St. George's Hospital, S.W.


*To the Editor of* The Hospital.

### St. George's Hospital.

*To the Editor of* The Hospital.

Sir,—In your note of our annual report, when commenting on the sale of Midland Stock, which realised £12,199, you say, "as the sale of stock was in itself more than sufficient to make up the deficiency, there must have been some further reason for the financial operation described in the paragraph quoted."

The financial operation in question was substituting £8,000 worth of Midland Stock for £8,423 (value) of "Stocks" received and retained in kind (as appears in the accounts), and the balance of the sale only slightly exceeds the difference between income and expenditure.—Yours faithfully,

F. J. Frankau, Deputy Treasurer.
St. George's Hospital, S.W., April 20, 1916.

*** The statement in the report was to the effect that a sum of £12,199 derived from sale of stock, plus £*x* legacies, plus £*y* bank overdraft, was used to meet the deficiency of £11,296 between ordinary income and expenditure. We called attention to this remarkable statement. The explanation now appears to be that a large part of the extraordinary income for the year, (£14,868), was used for current purposes, and for convenience stock received instead of cash was exchanged for other stock in the possession of the hospital, which latter was subsequently sold. Is this the case? Why the original statement was further complicated by the allusion to the £*y* bank overdraft is a mystery we could wish explained. —Ed. The Hospital.

